# Surface roughness and characteristics of CAD/CAM zirconia and glass ceramics after combined treatment procedures

**DOI:** 10.1186/s12903-022-02389-7

**Published:** 2022-11-23

**Authors:** Sibel Dikicier, Cumhur Korkmaz, Arzu Atay

**Affiliations:** Department of Prosthodontics, Hamidiye Faculty of Dentistry, University of Health Sciences, 34660 Uskudar Istanbul, Turkey

**Keywords:** Surface property, Dental air-abrasion, Dental acid etching, Er-YAG laser

## Abstract

**Background:**

The roughening of the inner surface of a fixed ceramic restoration is an important factor for the bonding process. The aim of this study is to investigate the effect of combined surface treatments (acid etching, air-abrasion and Er: YAG Laser) on surface roughness of CAD/CAM fabricated zirconia (ZrO_2_) and lithium-disilicate glass ceramics (LDS).

**Methods:**

Sixty ZrO_2_ (Ceramill Zi) and LDS (IPS e.max CAD) specimens, (5 mm in width, 5 mm in length and 1.5 mm in height) were fabricated using CAD/CAM and sintered according to the manufacturer’s instructions. All specimens subjected to three surface treatment combinations; etching with 4% hydrofluoric acide (HF), airborne-particle abrasion with 110-μm alumina (Al_2_O_3_) (AP) and Er:YAG laser (Er:YAG) (Group A—HF + AP; Group B—Er:YAG + AP, and Group C—Er:YAG + HF). Perthometer was used to measure the surface roughness of the specimens before and after the tretments.

**Results:**

Group A presented the highest Ra (LDS 0.81 ± 0.27 and ZrO_2_ 0.67 ± 0.21 after treatment) and Group C the lowest (LDS 0.45 ± 0.13 and ZrO_2_ 0.26 ± 0.07, after treatment). Compared with before treatment, the Ra were significantly different only in Group A both ZrO_2_ and LDS after treatment (*p* < 0.05). Qualitative SEM images suggested the surface topography of the ZrO_2_ was smoother than the LDS. Less surface changes were observed in the Er:YAG combined procedures than HF + AP.

**Conclusions:**

HF + AP was significantly succesful in modifying the ceramic surface. Er:YAG did not sufficiently promote the surface topography, even if combined with any other treatments. Overall, surface tretments on ZrO_2_ not easier than LDS.

## Introduction

Superior esthetic characteristics of all-ceramic restorations made them highly popular through the last decade. With the elimination of metal infrastructures, optimal distribution of reflected light and translucency is achieved which leads to a highly esthetic appearance and simulating the natural appearance of natural tooth [1, 2]. With these advantages all-ceramic restorations are indicated for fixed prostheses such as ceramic inlay/onlay restorations, partial/full crowns and bridges.

Fort the aim of the achieve better prosthetic results, it has developed to the use of restorations produced by the CAD/CAM system (computer-aided design/computer-aided manufacturing), which is an extensive technique in last decades. This technique allowed to manufacture the fixed restorations in a single session with excellent accuracy and adaptation [3, 4], using industrially manufactured ceramic blocks [5, 6]. Materials with different compositions and microstructures are available for CAD/CAM, such as lithium disilicate glass–ceramics and zirconium-oxide based polycrystalline ceramics which were most popular on the dental market [6]. Although there were many recent studies, these ceramics were considered in the present study due to their widespread clinical option.

Glass–ceramics composed of leucite or lithium disilicate as basic crystalline structure have become preferred material due to their advanced physical, chemical, and mechanical properties [7]. Lithium disilicate glass–ceramics (LDS) based on SiO_2_–Li_2_O materials system are particularly commercially successful in dental applications. LDS are processed into full-contour restorations for inlays/onlays, veneers, crowns, and fixed partial dentures either by heat-pressing techniques or computer-aided design/computer-aided manufacturing (CAD/CAM) [7, 8]. The CAD/CAM method does not require multiple firings and the blocks have several advantages, such as fast milling and increased fracture resistance [9].

When yttria-stabilized tetragonal zirconia polycrystal (YTZP) is subject to thermomechanical factors, transformation of its tetragonal to monoclinic phase occurs [10]. This transformation-toughening property is responsible for its high fracture resistance [11] making zirconium oxide suitable for use as a framework for fixed prostheses, resin bonded fixed prostheses, and dental implant abutments [12, 13].

Bonding procedure is crucial for clinical survival rates for all-ceramic restorations. The diffusion of monomers into the demineralized dentine matrix, followed by polymerization, assists the micromechanical connection over hybrid layer formation [14]. Likewise, the internal surface of the ceramic restoration must be modified to manage the micromechanical connection between the ceramic and the resin cement. A number of techniques had been reported to enhance the bond strength between luting cement and ceramic [2, 15]. Etching with hydrofloric acid (HF) provides a well established bonding between resin cements and lithium disilicate glass ceramics which is a popular synthetic glass ceramic with the higher results in esthetic and mechanical characteristics [16, 17]. The microstructure of this ceramic changes by dissociation of one of the glassy phases of ceramic [18, 19]. This phase is fused rather to form an convenient surface structure for bonding [20–22]. Dissolving of the glassy phase exposes lithium disilicate crystals which shows as retentive characters [23]. Additionally, silane primers can provide a chemical bond between resin and glass ceramic [24, 25].

It was reported that hydrofloric acid application for 1–3 min provides successful results in terms of adhesive retention, usually in concentrations ranging from 2.5 to 10% [26–28]. However, some researchers have centered their studies in investigating an alternative surface procedure for glass ceramic, by obtaining better adhesion [29]. In recent years, it was reported that methods such as sandblasting, or laser irradiation for surface treatments of glass ceramics may also provide an optimal adhesion [2, 30, 31].

Zirconium dioxide (ZrO_2_) cannot be roughed enough with acid etching due to absence of a glassy phase [10]. Another surface treatment suggested before bonding for ZrO_2_ is airborne-particle abrasion (AP) with aluminum oxide which creates an irregular topography with expanding surface area thereby increasing the bond strength of resin to ceramic [32].

Although AP is known to be effective method to surface roughness of ZrO_2_, some reports discuss about airborne particle abrasion and the possible long-term adverse effect of external erosion on the strength of ZrO_2_ [33, 34]. Tribochemical silica coating and/or laser treatment has been presented as an option to airborne particle abrasion, in a try to advance the surface conditions of ZrO_2_. The laser application as ceramic surface treatment is still a disputable subject in dentistry. For dentistry practices especially including surface conditioning on ZrO_2_ for obtaining the best bonding strength, erbium:yttrium aluminum garnet (Er:YAG) laser is recommended [31]. Previous in-vitro studies reported that various laser types can be used effectively for modifying the microstructural characteristics of ZrO_2_ [35–37].

Many surface treatment methods are valid to develop an efficient bonding for ceramic surfaces. There was not sufficient information about the combination of both surface treatments which to create better roughness on different all-ceramic restorations. Thus, the present study aimed to evaluate the effect of varying combined surface treatments such as: HF with AP, HF with Er:YAG, and AP with Er:YAG, on surface conditions of ZrO_2_ and lithium-disilicate glass ceramic. The rationale for testing CAD/CAM ceramic surfaces is based on the clinical situation where adhesive cementation is needed. The null hypothesis were tested that various surface treatment combinations would not affect the surface rougness on zirconia and lithium disilicate ceramics.

## Materials and methods

### Sample preparation and surface treatments

Thirty (5 × 5 × 1.5 mm) pre-sintered zirconium oxide (ZrO_2_) (Ceramill Zi; Amann Girrbach) and thirty (5 × 5 × 1.5 mm) lithium disilicate glass ceramic (LDS) (IPS e.max CAD, Ivoclar-Vivadent) block-shaped samples were fabricated according to the manufacturer’s instructions from CAD/CAM machineable blocks. The surfaces of the samples were finished with a 600–1200 grit metallographic paper with a polishing machine (Labpol 8–12, Extec). and then crystallized or sintered according to the manufacturer's instructions (with a temperature of 1600 °C for ZrO_2_ and 770 °C LDS). Glazing was not applied since it was purposed to evaluate the untreated inner surface of the ceramic. All specimens were divided into two groups according to the ceramic material (n = 30/ceramic material), where each group was further subdivided into three subgroups (n = 10/subgroup) according to the surface treatment type (Table 1). Samples in Subgroup A were etched with 4% hydrofluoric acid gel (Porcelain Etch Hydrofluoric Acid, Ultra-dent Products Inc.) for thirty seconds and sandblasted with air-abrasion (110 µm Al_2_O_3_) (Korox, 110#46,014; BEGO), in subgroup B; were treated with Er:YAG laser and sandblasted with air-abrasion (110 µm Al_2_O_3_), and in subgroup C; were treated with Er:YAG laser and etched with 4% hydrofluoric acid gel. After the airborne particle abrasion, samples were washed with drinking water for 1 min and ultrasonically cleaned in a water bath for 10 min and air dried.

**Table 1:** Test groups and surface treatments

Group	Treatment protocol
Group A	Etched with 4% hydrofluoric acid gel for 30 s, rinsed for 1 m, air dried for 10 s + and sandblasted with air-abrasion (110 µm Al_2_O_3_) for 20 s, with 4 bar pressure, distance of 10 mm
Group B	Er:YAG laser applied energy level was 150 mJ with 10-Hz frequency for 45 s, pulse width 300 μS + and sandblasted with air-abrasion (110 µm Al_2_O_3_) for 20 s, with 4 bar pressure, distance of 10 mm
Group C	Er:YAG laser applied energy level was 150 mJ with 10-Hz frequency for 45 s., pulse width 300 μS + and etched with 4% hydrofluoric acid gel for 30 s, rinsed for 1 m, air dried for 10 s

### Surface analysis and SEM procedure

Surface roughness of the samples was evaluated with Perthomether M2 (Mahr Co.) measuring device to scan the surface roughness with a microneedle, utilizing the surface roughness parameter (Ra). Three measurements were performed on the surface of each sample in following directions with a cutoff value of 0.25 mm (λc) and a speed of 0.1 mm/s, longitudinal, transversal, and oblique [38]. The surface profile was recorded and the mean Ra expressed in μm was determined.

The superficial topography was observed using scanning electron microscope (JSM-6 6400, JEOL). For this procedure, the samples were coated with a gold–palladium alloy spray and observed to evaluate the surface model. Photographs were taken at a magnification of 500 × /1000 × and used for comparison in surface smoothness.

### Statistical analysis

A descriptive analysis of the roughness data (Ra) were evaluated by using SPSS (Statistical Package for the Social Sciences, version 13.5, SPSS Inc.) to determine the mean and standard deviations. Comparisons within the groups for differences in surface roughness before and after treatment were performed using Wilcoxon signed rank test. The effects of combined surface treatment procedures on between ceramic groups were performed using one-way ANOVA. *p* value was set at ≤ 0.05.

## Results

The comparisons are presented in Table 2. According to surface analysis, among both Ra values, Group A (AP combined HF) presented a statistical difference (*p* < 0.05), in both ZrO_2_ and LDS specimens (Ra for ZrO_2_, 0.26/0.67, Ra for LDS, 0.34/0.81, before / after treatment, respectively). Group A also led to statistically significant higher Ra values in comparison to Group B (Er:YAG combined AP) and/or Group C (Er:YAG combined HF) (*p* < 0.05). None of the Er:YAG combination treatments of surface treatment groups (Group B and C) did not showed statistically difference in all surface treatment group specimens.

**Table 2:** Surface roughness values (Ra) of various surface treated all-ceramics

Group	Treatment protocol	Material	Mean(SD) Ra (μm)		p*
			Before treatment	After treatment	
Group A (n = 20)	HF + AP	Zirconium oxide	0.2(0.09)^a^	0.6(0.01)^b^	< 0.05
		Lithium disilicate	0.3(0.05)^a^	0.8(0.07)^c^	< 0.05
Group B (n = 20)	Er:YAG + AP	Zirconium oxide	0.2(0.05)^a^	0.50(0.07)^a^	> 0.05
		Lithium disilicate	0.36(0.09)^a^	0.4(0.09)^a^	> 0.05
Group C (n = 20)	Er:YAG + HF	Zirconium oxide	0.2(0.07)^a^	0.35(0.07)^a^	> 0.05
		Lithium disilicate	0.23(0.09)^a^	0.55(0.13)^a^	> 0.05

*Wilcoxon signed ranks test (*p* < 0.05)

Within any column and line means with the same superscript letters are not significantly different (*p* > 0.05). Different letters indicate a significantly difference among the groups (*p* < 0.05)

*HF* hydrofluoric acid etching, *AP* airborne particle abrasion, *Er:YAG* erbium:yttrium aluminum garnet laser treatment

Regardless of the type of surface treatment applied on the ceramic, the Ra values of LDS was slightly higher than that of ZrO_2_. It must be emphasized that the Ra values between ZrO_2_ and LDS specimens were similar within both treatment groups. Additionally we may indicate that all treatment protocols were better than the untreated specimens.

**Fig. 1:**
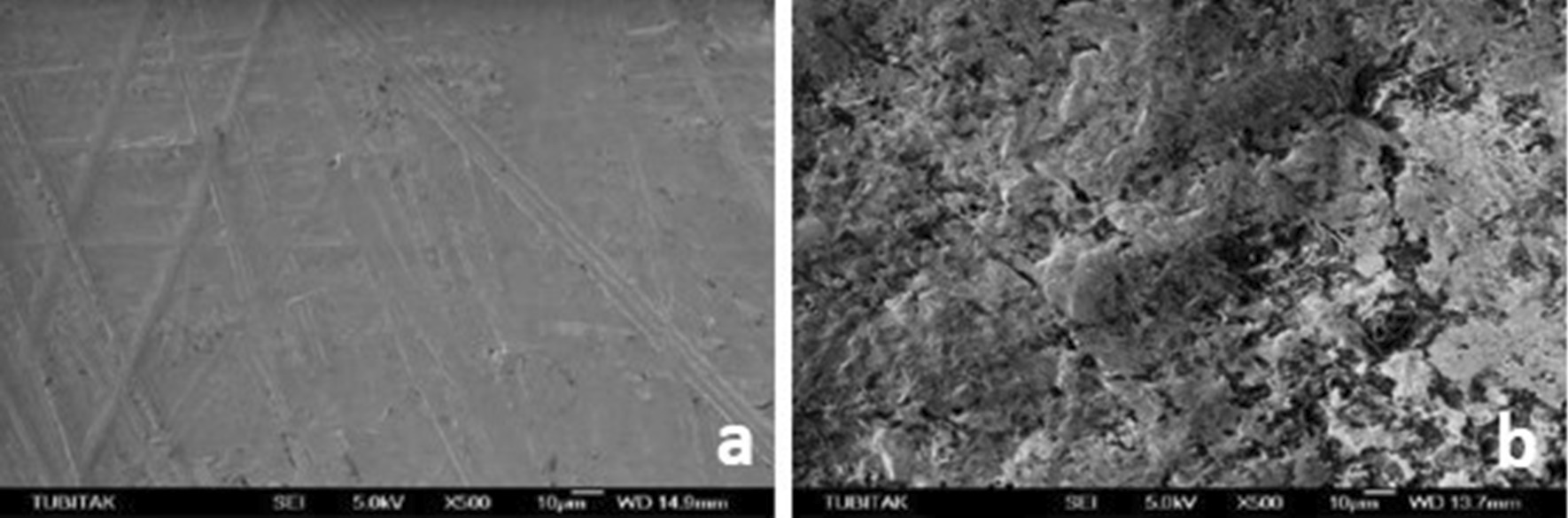
**a** SEM image of the zirconia samples before HF + AP, **b** after HF + AP

**Fig. 2:**
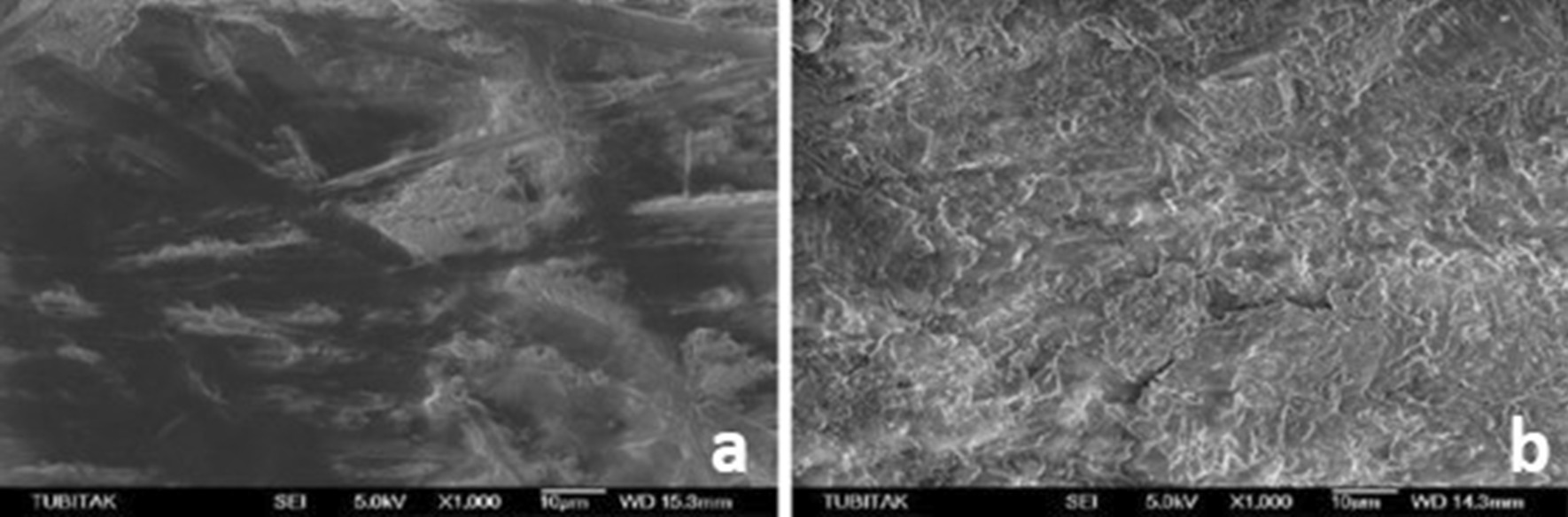
**a** SEM image of the zirconia samples before Er:YAG + AP, **b** after Er:YAG + AP

**Fig. 3:**
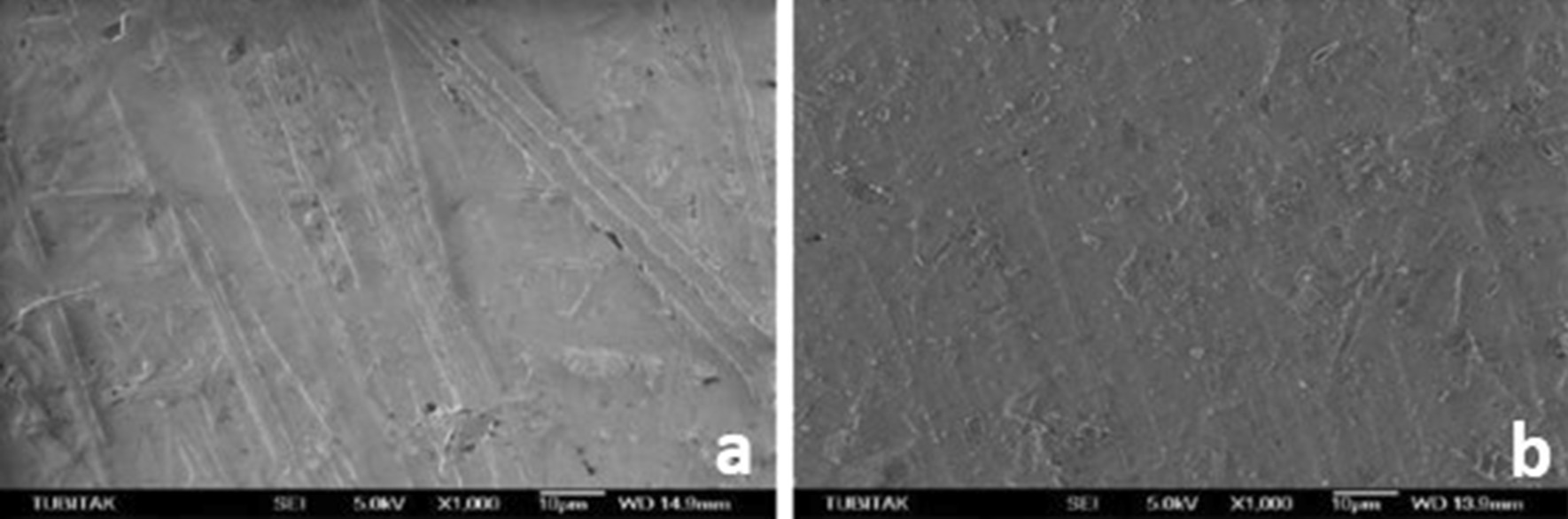
**a** SEM image of the zirconia samples before Er:YAG + HF, **b** after Er:YAG + HF

**Fig. 4:**
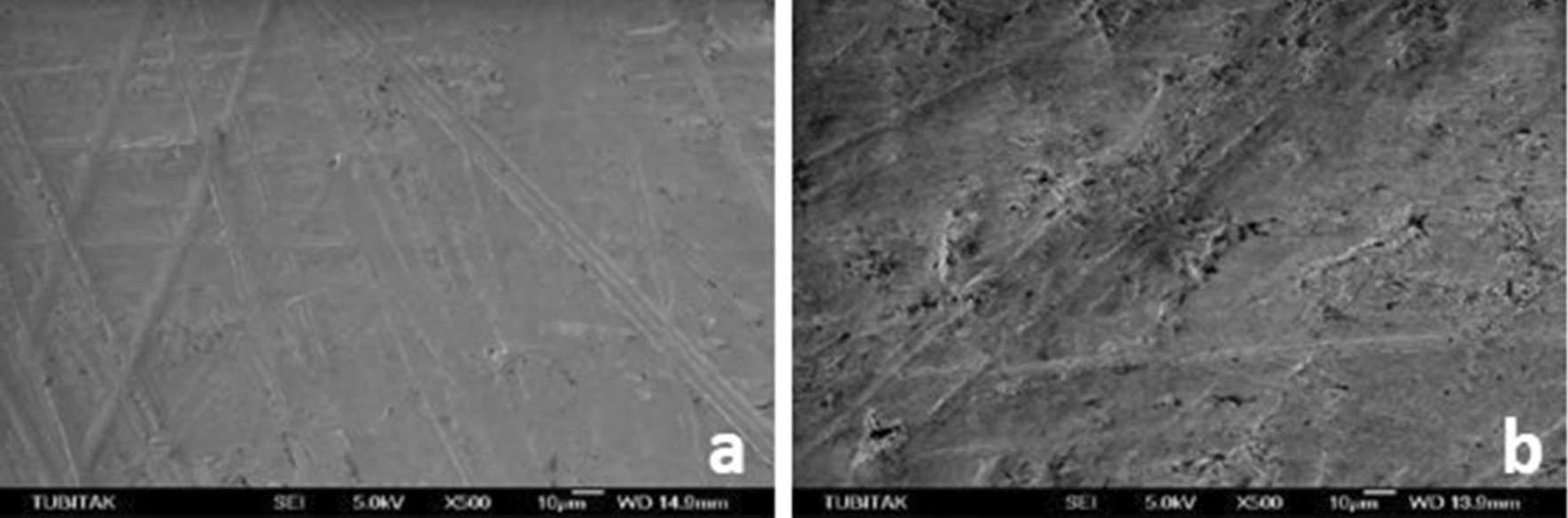
**a** SEM image of the lithium disilicate samples before HF + AP, **b** after HF + AP

**Fig. 5:**
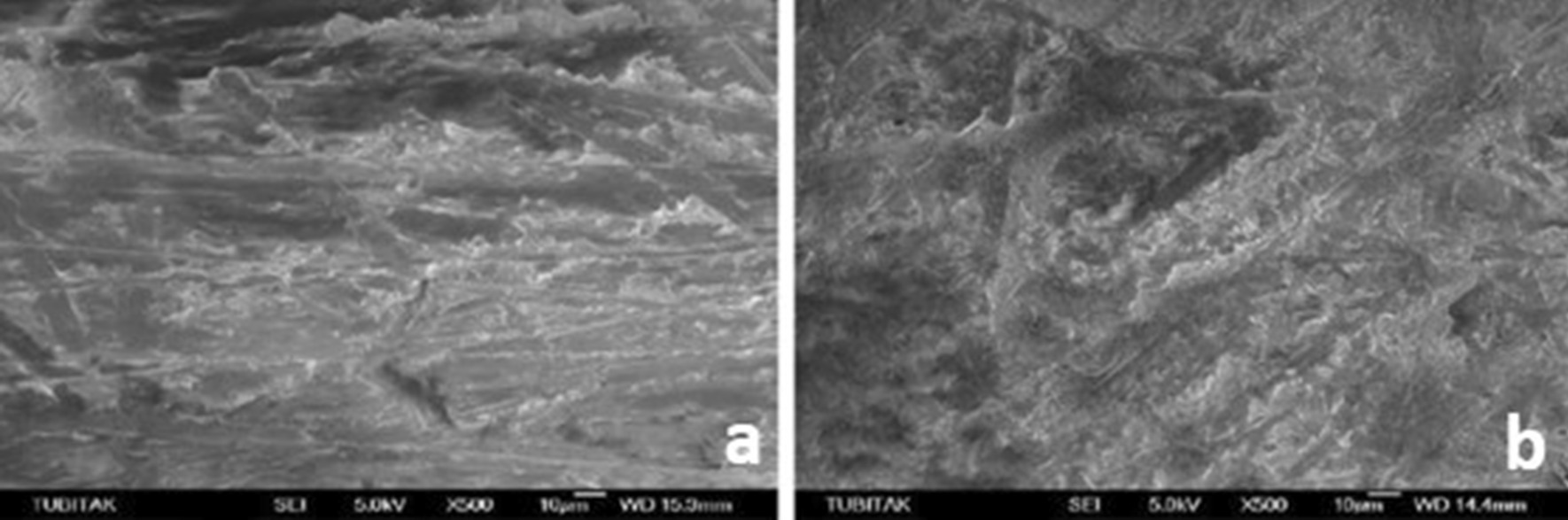
**a** SEM image of the lithium disilicate samples before Er:YAG + AP, **b** after Er:YAG + AP

**Fig. 6:**
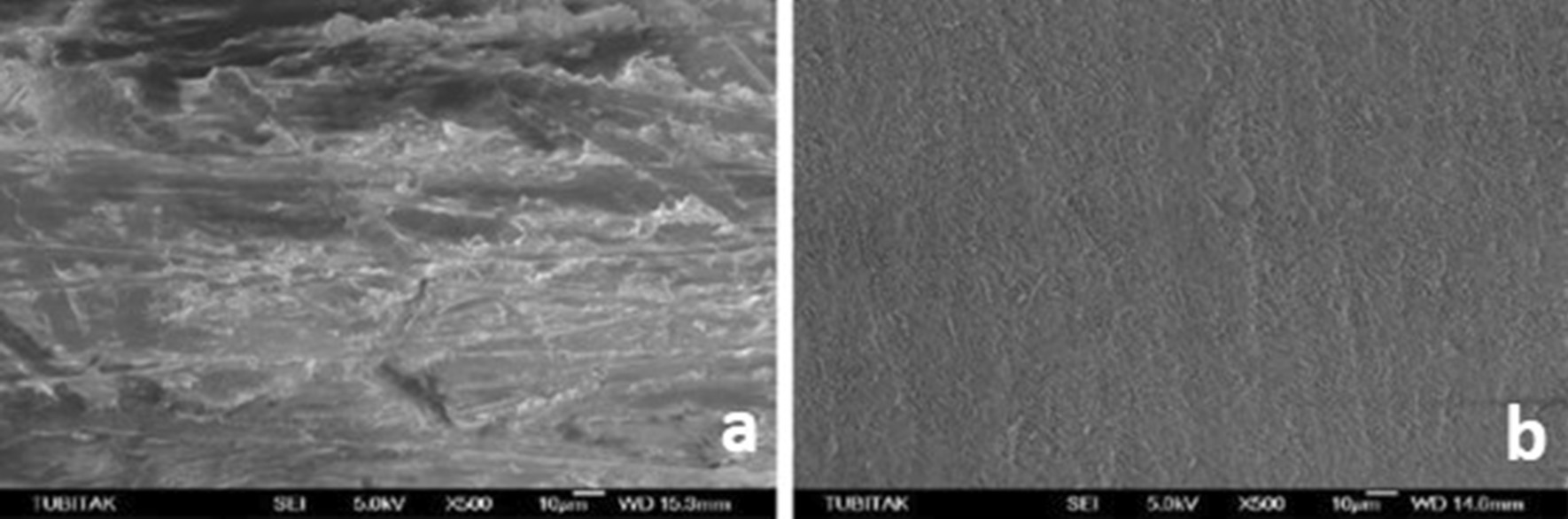
**a** SEM image of the lithium disilicate samples before Er:YAG + HF, **b** after Er:YAG + HF

SEM micrographs (500×/1000× magnification) showed different surface characteristics of specimens subjected to studied procedures. It emphasized that both experimental ceramic types presented the different topographical pattern before and after treatment and that HF and AP procedure modifies the materials surface attributes its external pattern (Figs. 1, 2), while Er:YAG combined conditions appears to few relate in such outcome (Figs. 3, 4, 5, 6).

## Discussion

This study investigated the surface parameters in LDS and ZrO_2_ all-ceramics by different surface treatments. The experimental design used for the etching, airborne particle abrasion and Er:YAG laser treatments were selected based on previous studies [23, 32, 39]. Surface roughness is one major perspective that describes the efficiency of pre-treatment procedures. For the analysis of the pretreated and treated surfaces of the ceramic specimens, Ra values was used as in many studies [40, 41], but there is no ideal clinically relevant amount of roughness known so far. The findings of this study required the partly rejection of the null hypothesis because changes observed in the surface topography of all-ceramics after surface treatment combinations. However, the proper selection of the surface treatment seemed to be a more important factor relation with ceramic type.

To achieve effective bonding between tooth surface and ceramic, mechanical retention, by surface roughening and microchemical connection with a silane agent are essential. The authors considered that topographic differences of the surface after etching, airborne particle abrasion and/or Er:YAG laser irradiation may have a great effect on adhesion strength. In the present study, among the study groups, statistically significant difference occurs between before and after surface treatment in Group A (HF + AP) when compared with the Group B (Er:YAG + AP) and C (Er:YAG + HF). Furthermore, Group B and C showed similar Ra values, in both ceramic types.

Various surface procedures have been valid to evolve suitable bonding surface to ZrO_2_ ceramic; such as airborne-particle abrasion, acid etching, tribochemical silica application [42, 43]. AP performed a prepared micro-retentive ZrO_2_ surface, increased the adhesion capacity, and improved the surface tension and wettability, hereby increasing the creation of cement-ceramic micromechanical connection [44]. Kim et al. [23] tested ZrO_2_ ceramics and found that airborne-particle abrasion or acid etching alone few affected to ensure dependable bond strength between resin and ZrO_2_ ceramics. Additionally, Anand et al. [45] supported the view that conventional methods did not obtain clinically sufficient bond strength values. These results encouraged our study to produce another prebonding surface treatment combinations.

In dental ceramics as LDS, containing glass particles, the surface roughness can be formed with HF for acceptable bonding, while the surface of ZrO_2_ ceramics is without glass phase; HF does not show any significant increase on bond strength [45]. Therefore, HF was showed a chemical benefit rather than mechanical benefit because previous studies demonstrated it ineffective at surface conditioning in ZrO_2_ ceramics [1, 2, 46]. In this study, while HF combination treatments indicated higher results for LDS ceramics, it also improved the surface characteristics of ZrO_2_ ceramic. This effect on ZrO_2_ was probably caused by the AP stage in the process.

The main structure of IPS e.max Press glass ceramic is shaped by prolonged glass crystals of LDS. Another phase is formed of lithium orthophosphate and a glass matrix encloses both crystalline structures. HF might reshape the glass and crystalline phase in this way composing rougness within the LDS crystals. In addition, the high level roughness improves the surface energy and the connection between the adhesive bonding and silane, thus supporting a micromechanical retention at the ceramic-resin interface [47]. The present study showed that 4% HF applied for 20 s combined with AP on the IPS e.max Press glass ceramic is suitable on the glass structure and therefore creates an irregular surface sufficient for bonding. Nevertheless, it should be focused that HF application on glass ceramics, remains as a primary important step on silanization procedures of glass–ceramics.

Although AP, not recommended by the manufacturers for LDS, few studies were presented in the literature on the surface roughness with AP [47, 48]. Gorman et al.[48] reported that AP procedure after etching did not damaged to the etched condition of the ceramic surface, while some abrasive particles may have embedded in the surface. Other study stated that of AP showed the creation of predictable microretentive grooves, but HF formed a microporous surface on SEM images [47]. However, it should be noted that, in the present study, in all combined surface treatment procedures, while the etching phase is more effective on surface roughness for LDS, airborne particle abrasion phase is more effective for ZrO_2_.

Many studies were focused that the AP procedure showed significantly higher Ra values on ZrO_2_ [39, 49]. It was also indicated that to avoid injuring ZrO_2_ and meantime provide optimal bond strength, AP should perform with proper particle size and jet pressure according to manufacturers recommedation.

Several researches have tested the surface treatment of ZrO_2_ to bonding mechanism with Er:YAG laser. Kunt et al. [31] reported no significant differences in surface roughness after laser treatment on zirconia surfaces, only CO_2_ laser irradiation technique were found succesfully and recommended as an alternate surface treatment to AP for ZrO_2_.

Our results showed that the Er:YAG/HF combination slightly changed the ZrO_2_ surface with the formation of limited number of micro-porousites. Nevertheless, Er:YAG /AP combined treatment is more effective due to the possible efficiency of AP phase on ZrO_2_. Zeidan et al. [38] indicated that there is a significant relation between the Er:YAG laser power capacity and the enhance of surface morphology of zirconia. It was considered that the effect of higher laser power on the ceramic surface revealed increased surface rougness without ceramic loss. The laser energy was selected as 150 mJ in our study, according to previous research and the potency of these factors was evaluated, and the results appeared similar to other surface treatments tested [32].

The tested LDS ceramic is the most preferable high translucent material in prosthodontics, and therefore there are a lot of informations about the surface parameters of this ceramic in the literature [47, 48, 50]. Laser irradiation is not commonly used to surface traetment for LDS. For this reason, there are few data about Er:YAG laser treatment on LDS and present study indicated that the Er:YAG treatment combinations is not as significantly successful as HF combinations on LDS surface. Our data supports a similar performance of the LDS to the ones that have been evaluated by previous studies [29, 51]. Nevertheless, more clinical and in vitro studies are necessary about surface parameters of dental ceramics, since there is still no sufficient data about effective laser type and applications modes.

In the present study, HF + AP combination was found to be effective technique for both ceramic types tested. Moreover, Cervino et al. [52] reported that sandblasting and acid-etching combination was a safe and successful method to modify the titanium dental implant surfaces. It may consider that HF + AP method with varying concentration and particle sizes is effective on metal surfaces in addition to ceramic surfaces. Future studies with dental material-comparative studies on this surface treatment combination may contribute to the clinic and manufacturing processes.

With the disadvantages of ceramic brittleness and new developments in adhesion technology, new materials such as, polyether ether ketone (PEEK) which is a thermoplastic resin polimer, and glass-fiber blocks, which is reinforced resin composite, may become popular as a framework material in prosthetic restorations [53]. It is also advantages that they can be produced with CAD/CAM technique and surface treatment can be applied.

According to the results of our study, all ceramic specimens demonstrated irregular surface after combined surface treatments applied. However, it may be stated that surface roughness is only a portion of the adhesion mechanism. Study limitations include the reality that are the comparison of alone and combined surface procedures together and testing with the bonding process. Another potential limitation of this in-vitro study is that the clinical situation cannot be completely represented. Therefore, further extensive in vitro and/or in vivo studies are necessary which consist adhesion procedure to approve the results of this in vitro study.

## Conclusion

Within the limitations of this in vitro study the following conclusions can be drawn:

- Only the HF + AP combined treatment succeeded on surface rougness of ZrO_2_ and LDS ceramic surfaces.
- Er:YAG + HF and/or Er:YAG + AP combined treatments did not significantly increase the surface rougness of the both ceramics tested.
- LDS showed higher Ra values than ZrO_2_ regardless of the surface procedure.
- The use of 4% HF acid etching + AP with 110 µm Al_2_O_3_ resulted in significantly higher surface roughness on to both LDS ZrO_2_ ceramics. These results may indicate improved bond strength.
- Conventional techniques such as HF and AP, alone or combined, still appeared to assume a more effective role in the surface treatment of the related ceramic types. Thus, the use of these techniques may bring succesful bonding benefits to restorations clinically.

## Data Availability

All data generated or analysed during this study are included in this published article.

## References

[1] Ozcan M, Vallittu PK (2003). Effect of surface conditioning methods on the bond strength of luting cement to ceramics. Dent Mater.

[2] Borges G, Sophr AN, de Goes MF, Sobrinho LC, Chan DC (2003). Effect of etching and airborne particle abrasion on the microstructure of different dental ceramics. J Prosthet Dent.

[3] Ludovichetti FS, Trindade FZ, Werner A, Kleverlaan CJ, Fonseca RG (2018). Wear resistance and abrasiveness of CAD-CAM monolithic materials. J Prosthet Dent.

[4] Papadiochou S, Pissiotis AL (2018). Marginal adaptation and CAD-CAM technology: a systematic review of restorative material and fabrication techniques. J Prosthet Dent.

[5] Winter A, Schurig A, Rasche E, Rösner F, Kanus L, Schmitter M (2019). The flexural strength of CAD/CAM polymer crowns and the effect of artificial ageing on the fracture resistance of CAD/CAM polymer and ceramic single crowns. J Mater Sci Mater Med.

[6] Lima CM, Silva NRD, Martins JD, Miranda JS, Tanaka R, Souza ROAE, Leite FPP (2021). Effect of different surface treatments on the biaxial flexure strength, Weibull characteristics, roughness, and surface topography of bonded CAD/CAM silica-based ceramics. Dent Mater.

[7] Deng BH, Luo J, Harris JT, Smith CM, McKenzie ME (2020). Toughening of Li_2_O-2SiO_2_ glass-cramics induced by intriguing deformation behavior of lithium disilicate nanocrystal. J Am Ceram Soc.

[8] Luangruangrong P, Cook NB, Sabrah AH, Hara AT, Bottino MC (2014). Influence of full-contour zirconia surface roughness on wear of glass-ceramics. J Prosthodont.

[9] Vasiliu RD, Porojan SD, Bîrdeanu MI, Porojan L (2020). Effect of thermocycling, surface treatments and microstructure on the optical properties and roughness of CAD-CAM and heat-pressed glass ceramics. Materials (Basel).

[10] Al-Amleh B, Lyons K, Swain M (2010). Clinical trials in zirconia: a systematic review. J Oral Rehabil.

[11] Aurélio IL, Marchionatti AM, Montagner AF, May LG, Soares FZ (2016). Does air particle abrasion affect the flexural strength and phase transformation of Y-TZP? A systematic review and meta-analysis. Dent Mater.

[12] Martins FV, Mattos CT, Cordeiro WJB, Fonseca EM (2019). Evaluation of zirconia surface roughness after aluminum oxide airborne-particle abrasion and the erbium-YAG, neodymium-doped YAG, or CO_2_ lasers: a systematic review and meta-analysis. J Prosthet Dent.

[13] Passos SP, Nychka JA, Major P, Linke B, Flores-Mir C (2015). In vitro fracture toughness of commercial Y-TZP ceramics: a systematic review. J Prosthodont.

[14] Nakabayashi N, Kojima K, Masuhara E (1982). The promotion of adhesion by the infiltration of monomers into tooth substrates. J Biomed Mater Res.

[15] Bottino MA, Valandro LF, Scotti R, Buso L (2005). Effect of surface treatments on the resin bond to zirconium based ceramic. Int J Prosthodont.

[16] Abuelenain DA, Linjawi AI, Alghamdi AS, Alsadi FM (2021). The effect of various mechanical and chemical surface conditioning on the bonding of orthodontic brackets to all-ceramic materials. J Dent Sci.

[17] de Kok P, Kleverlaan CJ, de Jager N, Kuijs R, Feilzer AJ (2015). Mechanical performance of implant-supported posterior crowns. J Prosthet Dent.

[18] Guarda GB, Correr AB, Goncalves LS, Borges GA, Sinhoreti MAC, Correr-Sobrinho L (2013). Effect of surface treatments, thermocycling, and cyclic loading on the bond strength of a resin cement bonded to a lithium disilicate glass ceramic. Oper Dent.

[19] Kato H, Matsumura H, Tanaka T, Atsuta M (1996). Bond strength and durability of porcelain bonding systems. J Prosthet Dent.

[20] Kukiattrakoon B, Thammasitboon K (2007). The effect of different etching times of acidulated phosphate fluoride gel on the shear bond strength of high-leucite ceramics bonded to composite resin. J Prosthet Dent.

[21] Yen TW, Blackman RB, Baez RJ (1993). Effect of acid etching on the flexural strength of a feldspathic porcelain and a castable glass ceramic. J Prosthet Dent.

[22] Holand W, Schweiger M, Frank M, Rheinberger V (2000). A comparison of the microstructure and properties of the IPS Empress 2 and the IPS Empress glass-ceramics. J Biomed Mater Res.

[23] Kim BK, Bae HE, Shim JS, Lee KW (2005). The influence of ceramic surface treatments on the tensile bond strength of composite resin to all-ceramic coping materials. J Prosthet Dent.

[24] Murillo-Gomez F, De Goes MF (2017). Effect of different silane treatments on long-term bonding between non-etched glass-ceramic and resin cement. Int J Dent Sci.

[25] Blatz MB, Sadan A, Kern M (2003). Resin-ceramic bonding: a review of the literature. J Prosthet Dent.

[26] Pospiech P (2002). All-ceramic crowns:bonding or cementing?. Clin Oral Invest.

[27] Chen JH, Matsumura H, Atsuta M (1998). Effect of different etching periods on the bond strength of a composite resin to a machinable porcelain. J Dent.

[28] Wolf DM, Powers JM, O’Keefe KL (1993). Bond strength of composite to etched and sandblasted porcelain. Am J Dent.

[29] Viskic J, Jokic D, Jakovljevic S, Bergman L, Ortolan SM, Mehulic K (2018). Scanning electron microscope comparative surface evaluation of glazed-lithium disilicate ceramics under different irradiation settings of Nd:YAG and Er:YAG lasers. Angle Orthodont.

[30] Amaral R, Ozcan M, Bottino MA, Valandro LF (2006). Microtensile bond strength of a resin cement to glass infiltrated zirconia-reinforced ceramic: the effect of surface conditioning. Dent Mater.

[31] Ergun Kunt G, Duran I (2018). Effect of laser treatments on surface roughness of zirconium oxide ceramics. BMC Oral Health.

[32] Qeblawi DM, Munoz CA, Brewer JD, Monaco EA (2010). The effect of zirconia surface treatment on flexural strength and shear bond strength to a resin cement. J Prosthet Dent.

[33] Zhang Y, Lawn BR, Malament KA, Van Thompson P, Rekow D (2006). Damage accumulation and fatigue life of particle-abraded ceramics. Int J Prosthodont.

[34] Zhang Y, Lawn BR, Rekow ED, Van Thompson P (2004). Effect of sandblasting on the long-term performance of dental ceramics. J Biomed Mater Res B Appl Biomater.

[35] Kirmali O, Kustarci A, Kapdan A (2015). Surface rougness and morphological changes of zirconia: Effect of different surface treatment. Niger J Clin Pract.

[36] Spohr AM, Borges GA, Júnior LH, Mota EG, Oshima HM (2008). Surface modification of In-Ceram Zirconia ceramic by Nd: YAG laser, Rocatec system, or aluminum oxide sandblasting and its bond strength to a resin cement. Photomed Laser Surg.

[37] Cavalcanti AN, Pilecki P, Foxton RM (2009). Evaluation of the surface roughness and morphologic features of Y-TZP ceramics after different surface treatments. Photomed Laser Surg.

[38] Zeidan LC, Esteves CM, Oliveira JA, Brugnera A, Cassoni A, Rodrigues JA (2018). Effect of different power settings of Er, Cr:YSGG laser before or after tribosilicatization on the microshear bond strength between zirconia and two types of cements. Lasers Med Sci.

[39] Zhao P, Yu P, Xiong Y, Yue L, Arola D, Gao S (2020). Does the bond strength of highly translucent zirconia show a different dependance on the airborne-particle abrasion parameters in comparison to conventional zirconia?. J Prosthodont Res.

[40] Mota EG, Smidt LN, Fracasso LM, Burnett LH, Spohr AM (2017). The effect of milling and postmilling procedures on the surface roughness of CAD/CAM materials. J Esthet Restor Dent.

[41] Abdullah AO, Hui Y, Sun X, Pollington S, Muhammed FK, Liu Y (2019). Effects of different surface treatments on the shear bond strength of veneering ceramic materials to zirconia. J Adv Prosthodont.

[42] Yang B, Barloi A, Kern M (2010). Influence of airabrasion on zirconia ceramic bonding using an adhesive composite resin. Dent Mater.

[43] Al-Dohan HM, Yaman P, Dennison JB, Razzoog ME, Lang BR (2004). Shear strength of core-veneer interface in bi-layered ceramics. J Prosthet Dent.

[44] Attia A, Kern M (2011). Long-term resin bonding to zirconia ceramic with a new universal primer. J Prosthet Dent.

[45] Anand S, Ebenezar AVR, Anand N, Rajkumar K, Mahalaxmi S, Srinivasan N (2015). Microshear bond strength evaluation of surface pretreated zirconia ceramics bonded to dentin. Eur J Dent.

[46] Dérand P, Dérand T (2000). Bond strength of luting cements to zirconium oxide ceramics. Int J Prosthodont.

[47] Maruo Y, Nishigawa G, Irie M, Yoshihara K, Matsumoto T, Minagi S (2017). Does acid etching morphologically and chemically affect lithium disilicate glass ceramic surfaces?. J Appl Biomater Funct Mater.

[48] Gorman CM, de Faoite D, Flannery D, Ratajczak M, Kelly T, Stanton KT (2019). Alteration of the intaglio surface of lithium disilicate glass-ceramic. J Prosthet Dent.

[49] Martins SB, Abi-Rached FO, Adabo GL, Baldissara P, Fonseca RG (2019). Influence of particle and air-abrasion moment on Y-TZP surface characterization and bond strength. J Prosthodont.

[50] Souza KB, Moura DMD, Silva SEGD, Araujo GM, Pinto RAS, Leite FPP, Ozcan M, Souza ROAE (2020). Effect of different surface treatments and multimode adhesive application on the Weibull characteristics, wettability, surface topography and adhesion to CAD/CAM lithium disilicate ceramic. J Appl Oral Sci.

[51] Kara HB, Dilber E, Koc O, Ozturk AN, Bulbul M (2012). Effect of different surface treatments on roughness of IPS Empress 2 ceramic. Lasers Med Sci.

[52] Cervino G, Fiorillo L, Iannello G, Santonocito D, Risitano G, Cicciù M (2019). Sandblasted and acid etched titanium dental implant surfaces systematic review and confocal microscopy evaluation. Materials (Basel).

[53] Fiorillo L, D’Amico C, Turkina AY, Nicita F, Amoroso G, Risitano G (2020). Endo and exoskeleton: new technologies on composite materials. Prosthesis.

